# Depiction of Aging-Based Molecular Phenotypes With Diverse Clinical Prognosis and Immunological Features in Gastric Cancer

**DOI:** 10.3389/fmed.2021.792740

**Published:** 2022-02-01

**Authors:** Fang He, Huan Ding, Yang Zhou, Yuanzhen Wang, Juan Xie, Shaoqi Yang, Yongzhao Zhu

**Affiliations:** ^1^General Hospital of Ningxia Medical University, Yinchuan, China; ^2^Graduate School, Ningxia Medical University, Yinchuan, China

**Keywords:** gastric cancer, aging-relevant genes, molecular phenotype, prognosis, tumor immune microenvironment, immunogenomic characteristics

## Abstract

**Objective:**

Aging acts as a dominating risk factor for human cancers. Herein, we systematically dissected the features of transcriptional aging-relevant genes in gastric cancer from multiple perspectives.

**Methods:**

Based on the transcriptome profiling of prognostic aging-relevant genes, patients with gastric cancer in The Cancer Genome Atlas (TCGA) stomach adenocarcinoma (TCGA-STAD) cohort were clustered with a consensus clustering algorithm. Mutational landscape and chemotherapeutic responses were analyzed and immunological features (immunomodulators, immune checkpoint molecules, cancer immunity cycle, and tumor-infiltrating immune cells) were systematically evaluated across gastric cancer. Weighted gene co-expression network (WGCNA) was conducted for screening aging molecular phenotype-relevant genes, and key genes were identified with Molecular Complex Detection (MCODE) analyses. Expressions of key genes were examined in 20 paired tumors and controls with RT-qPCR and Western blotting. Proliferation and apoptosis were investigated in two gastric cancer cells under MYL9 deficiency.

**Results:**

Three aging-based molecular phenotypes (namely, C1, C2, and C3) were conducted in gastric cancer. Phenotype C1 presented the most prominent survival advantage and highest mutational frequencies. Phenotype C2 indicated low responses to sorafenib and gefitinib, while C3 indicated low responses to vinorelbine and gemcitabine. Additionally, phenotype C2 was characterized by enhanced immune and stromal activation and an inflamed tumor microenvironment. Seven aging molecular phenotype-relevant key genes (ACTA2, CALD1, LMOD1, MYH11, MYL9, MYLK, and TAGLN) were identified, which were specifically upregulated in tumors and in relation to dismal prognosis. Among them, MYL9 deficiency reduced proliferation and enhanced apoptosis in gastric cancer cells.

**Conclusion:**

Collectively, aging-based molecular subtypes may offer more individualized therapy recommendations and prognosis assessment for patients in distinct subtypes.

## Introduction

Gastric cancer ranks the sixth most frequent malignancy as well as the fifth major cause of cancer death across the globe (1). When diagnosed at an advanced stage, patients' 5-year overall survival rate is merely 5% (1). As a heterogeneous disease, it has the features of diverse histological and molecular subtypes (2). At present, according to the morphology, differentiation, and cohesion of gland cells, gastric cancer is histopathologically classified as intestinal and diffuse (3). Genomic analyses have become the major methodology applied in international efforts for discovering novel biological targets in gastric cancer (4). It is fundamental to unravel the complicated biology underlying gastric cancer etiology and development for overcoming the highly heterogeneous malignancy.

Accumulated pieces of evidence have uncovered the implication of tumor-associated structures and activated signaling pathways both in tumor cells and in the tumor microenvironment (5). Aging is a complicated process primarily categorized by a reduction in tissues, cells, and organ functions as well as an elevated risk of mortality, which acts as a dominant risk factor of diverse fatal malignancies, especially cancers (6). This process presents prominent correlations to telomere attrition, mitochondrial dysfunction, DNA injury, impaired immune system, and the like (7). Nevertheless, the specific mechanisms involved in aging are still indistinct. Transcriptomic studies have identified abundant human aging-relevant genes (8). The human aging genome resource (HAGR) project offers a powerful set of aging-specific network features, which reveals aging-relevant gene signatures as network hubs through comprehensive analyses of biology and genetics of the human aging process (8). Cellular senescence is a permanent state of stagnant replication of proliferating cells as well as a sign of aging (9). Senescent tumor cells triggered by tumorigenesis may lead to cell cycle arrest, as an antitumor mechanism (10). Nevertheless, senescent cells surrounding tumor cells generate opposite results as well as present prominent correlations with senescence-associated secretory phenotype factor secretions (11). Moreover, senescence displays two-tier influences upon cancer immunity (12, 13). Aging-relevant gene signatures exert critical functions in modulating cellular senescence, not only inhibiting tumor progression through modulating senescence of cancer cells but also promoting malignant progression of cancers and dismal clinical outcomes (14). Nevertheless, there is still a lack of systematic analyses of aging-relevant genes during gastric carcinogenesis. Herein, we identified three aging-based molecular phenotypes that offered more individualized therapy options and prognosis prediction for gastric cancer patients.

## Materials and Methods

### Retrieval and Preprocessing of Genome-Relevant Data and Clinical Information

Raw RNA-seq data [Fragments Per Kilobase Million (FPKM) value] and relevant clinicopathological characteristics for The Cancer Genome Atlas (TCGA) stomach adenocarcinoma (TCGA-STAD) cohort containing 443 patients with gastric cancer were curated from Genomic Data Commons (GDC) data portal (https://portal.gdc.cancer.gov) utilizing TCGAbiolinks package (15). Additionally, normalized microarray expression profiling of 433 patients with gastric cancer in the GSE84437 cohort was curated from the Gene Expression Omnibus (GEO) repository (https://www.ncbi.nlm.nih.gov/gds/) (16). The detailed information of patients with gastric cancer in TCGA and GSE84437 cohorts is listed in Supplementary Table 1. The expression profiling (FPKM values) of the TCGA-STAD dataset was transformed into transcripts per kilobase million (TPMs). In total, 307 aging-relevant genes (Supplementary Table 2) were curated from the HAGR (https://genomics.senescence.info/) (8). Molecular subtypes [genome stable (GS), microsatellite instability (MSI), EBV infection, and chromosomal instability (CIN)] of gastric cancer samples were retrieved from Liu et al. (17). Somatic mutation data [Mutation Annotation Format (MAF) format] of 433 patients with gastric cancer on the basis of the whole-exome sequencing platforms were curated from the TCGA project. Mutational types and frequencies of genes were analyzed as well as visualized utilizing the maftools package (18). Tumor mutation burden (TMB) was defined as the entire number of non-synonymous variations within the coding regions per megabase (19). In addition, copy number alteration (CNA) data were retrieved from GDAC Firehose (https://gdac.broadinstitute.org), and prominent amplification and deletion across the whole genome were identified with GISTIC2.0 (20). Somatic copy-number alterations (SCNAs) and homologous recombination deficiency (HRD) across gastric cancer specimens were also curated from Davoli et al. (21).

### Molecular Characterization for Subtypes

Tumors with qualitatively diverse aging-relevant gene expressions were clustered utilizing hierarchical agglomerative clustering on the basis of Euclidean distance as well as Ward's linkage. Unsupervised clustering method (K-means) was utilized for identifying aging-related molecular phenotypes as well as classifying samples for subsequent analyses. Through consensus clustering algorithm, the number of clusters was determined using TCGA-STAD and GSE84437 cohorts for assessing the stability of the identified molecular phenotypes. This procedure was presented through adopting the ConsensuClusterPlus package as well as repeated 50 times for ensuring the accuracy regarding this classification (22).

### Gene Set Variation Analysis (GSVA)

Gene set variation analysis, a non-parametric and unsupervised gene set enrichment algorithm, may infer the enrichment scores of specific pathways or signatures on the basis of transcriptomic profiling (23). The 50 hallmarks of gene signatures were collected from the Molecular Signatures Database (MSigDB) project (24). Moreover, the gene sets of other relevant biological processes were curated from Mariathasan et al. containing CD8 T effectors, DNA damage repair, pan-fibroblast TGF-β response signature (Pan-F-TBRS), antigen-processing machinery, immune checkpoint, epithelial-mesenchymal transition (EMT) markers, FGFR3-related genes, angiogenesis, Fanconi anemia, WNT targets, cell cycle regulators, and the like (25). Utilizing single sample gene set enrichment analysis (ssGSEA) from the GSVA package, gene sets of hallmarks and other relevant biological processes were chosen for presenting quantifications of pathway activity.

### Estimation of Chemotherapeutic Response

Chemotherapeutic sensitivity in cancer cells, as well as molecular markers of chemotherapeutic response profiles, were curated from the largest publicly available pharmacogenomics project: the Genomics of Drug Sensitivity in Cancer (GDSC; https://www.cancerrxgene.org/) (26). Four commonly applied chemotherapeutic agents, sorafenib, gefitinib, vinorelbine, and gemcitabine, were chosen. The prediction procedure was implemented *via the* pRRophetic package (27). The half-maximal inhibitory concentration (IC50) values were estimated with the ridge regression method, and the prediction accuracy was assessed through 10-fold cross-verification.

### Evaluation of Tumor Immune Microenvironment and Immunogenomic Characteristics

Immunological features of the tumor immune microenvironment contained the expression profiles of immunomodulatory factors and immune checkpoint molecules, the activity of the cancer immunity cycle, and infiltrations of immune cells. In total, 122 immunomodulatory factors comprising MHC, receptor, chemokine, and immune stimulator were curated from Sokolov et al. (28). Immune checkpoint molecules with therapeutic potential were collected from Auslander et al. (29). The cancer immunity cycle uncovers antitumor immune response, and the activity of each step determines the fate of tumor cells (30). Here, the activity of each step was quantified with ssGSEA on the basis of the expression profiling of individual specimens. Thereafter, the ssGSEA algorithm was developed for quantifying the abundance of lymphocytes within the tumor immune microenvironment utilizing bulk RNA-seq profiles. Through Estimation of Stromal and Immune cells in Malignant Tumor tissues using Expression data (ESTIMATE), immune and stromal contents (immune and stromal scores and tumor purity) were inferred across gastric cancer specimens (31). Tumor tissues with abundant immune cell infiltration represented an increased immune score and a decreased level of tumor purity.

### Quantification of Gene Expression-Based Stemness Index (MRNAsi)

Through the one-class logistic regression (OCLR) method, the stemness index was calculated on the basis of transcriptome profiling of normal PSCs (32). The stemness signatures were generated with the OCLR algorithm (28). Thereafter, this study estimated Spearman's correlation between the weight vector of the stemness signatures and mRNA expression across gastric cancer. Eventually, the stemness index was mapped onto the range of 0 to 1 utilizing a linear conversion, which subtracted the minimum as well as separated through the maximal correlation coefficient. The stemness index produced from transcriptome profiling was defined as mRNAsi.

### Weighted Gene Co-expression Network Analysis (WGCNA)

Weighted gene co-expression network analysis was presented for identifying underlying co-expression modules that were prominently correlated with aging-associated molecular phenotypes. The soft-thresholding for the scale-free network was identified. The topological overlap matrix similarity was adopted for the evaluation of the distance between gene pairs. Furthermore, hierarchical clustering analyses with mean and dynamic methods were utilized for building the clustering tree as well as classifying the gene signatures into diverse modules. Following merging the initial modules in line with their similarity, functional modules were eventually conducted. Spearman's correlation coefficient, as well as matched *p*-value between aging-associated molecular phenotypes and functional modules, was determined through cor function. For each module, gene significance (GS) and module membership (MM) were calculated. Genes with GS > 0.5 and MM > 0.8 were utilized as aging phenotype-relevant genes. Through the Search Tool for the Retrieval of Interacting Genes/Proteins (STRING) tool (33), protein-protein interaction (PPI) analysis of aging phenotype-relevant genes was carried out. Molecular Complex Detection (MCODE) (34), a plugin in Cytoscape software (35), was used for screening the significant modules of the PPI network in line with the filtrating criteria of degree cutoff = 2, node score cutoff = 0.2, k-core = 2, and depth from depth = 100.

### Patients and Specimens

In total, 20 patients with gastric cancer were recruited at the General Hospital of Ningxia Medical University. Adjacent gastric tissues (3–6 cm from the tumor). Tumor tissues and adjacent non-cancerous gastric tissues (>5 cm from the edge of tumor tissues) were harvested during surgical resection. The inclusion criteria included: (1) patients pathologically diagnosed with gastric cancer and (2) patients who did not experience radio- and/or adjuvant chemotherapy prior to surgery. The exclusion criteria included: (1) patients previously diagnosed with other malignancies; (2) patients who were previously treated with radio- or adjuvant chemotherapy; and (3) patients who died within 4 weeks of this surgery. All specimens were frozen in liquid nitrogen at once following collection and were stored at −80°C before usage. This study was conducted in accordance with the guidance of the Declaration of Helsinki. The protocol gained the approval of the Institutional Ethical Committee of General Hospital of Ningxia Medical University (Approval No. 2020-031). All participants signed an informed consent form prior to our study.

### Real-Time Quantitative Reverse Transcription PCR

Tissues or cells were lysed with RNAiso plus (Takara, Japan). Thereafter, RNA extraction was presented with the phenol-chloroform/isopropanol method. The cDNA was prepared through PrimeScript RT reagent kits as well as a gDNA eraser. About 20 μl qPCR system was prepared, followed by analysis with GoTaq qPCR Master Mix. The relative mRNA expressions were quantified with the 2^−ΔΔCt^ method, with GAPDH as a control. The primer sequences are listed in Table 1.

**Table 1:** Primer sequences used for RT-qPCR.

**Gene**	**Sequence (5′-3′)**
MYL9	F: TCTTCGCAATGTTTGACCAGT
	R: GTTGAAAGCCTCCTTAAACTCCT
ACTA2	F: AAAAGACAGCTACGTGGGTGA
	R: GCCATGTTCTATCGGGTACTTC
TAGLN	F: AGTGCAGTCCAAAATCGAGAAG
	R: CTTGCTCAGAATCACGCCAT
MYH11	F: CGCCAAGAGACTCGTCTGG
	R: TCTTTCCCAACCGTGACCTTC
LMOD1	F: GTAAAAGGGGAGCGTAGGAAC
	R: CTCGGGTGTTTTGGTCTTGCT
CALD1	F: TGGAGGTGAATGCCCAGAAC
	R: GAAGGCGTTTTTGGCGTCTTT
MYLK	F: CCCGAGGTTGTCTGGTTCAAA
	R: GCAGGTGTACTTGGCATCGT
GAPDH	F: CTGGGCTACACTGAGCACC
	R: AAGTGGTCGTTGAGGGCAATG

### Western Blotting

Tissues or cells were lysed with RIPA buffer plus protease inhibitor cocktail. Following separation *via* electrophoresis in SDS/PAGE gel, the protein was transferred onto PVDF membranes. The membrane was blocked in PBS-T buffer in supplements of 5% milk/BSA lasting 2 h and presented the incubation with primary antibody targeting MYL9 (1:500; 15354-1-AP; Proteintech, China), ACTA2 (1:1000; 23081-1-AP; Proteintech, China), TAGLN (1:300; 15502-1-AP; Proteintech, China), MYH11 (1:1000; 18569-1-AP; Proteintech, China), LMOD1 (1:500; 15117-1-AP; Proteintech, China), CALD1 (1:2000; 20887-1-AP; Proteintech, China), MYLK (1:500; 21642-1-AP; Proteintech, China), and β-actin (1:5000; 20536-1-AP; Proteintech, China) overnight at 4°C. Following incubation by horseradish peroxidase-labeled HRP-coupled secondary antibodies lasting 1 h, the protein band was visualized with an ECL detection reagent.

### Cell Culture

Two gastric cancer cell lines (MGC-803 and BGC-823) were acquired from the Cell Bank of the Chinese Academy of Sciences (Shanghai, China). The cells were cultivated in the Dulbecco's modified Eagle's medium (DMEM; Gibco, United States) with supplements of 10% fetal bovine serum (FBS) as well as 1% antibiotics penicillin/streptomycin. Moreover, all cells were fostered in an incubator with 5% CO_2_ at 37°C.

### Transfection

For generating MYL9-knockdown clones, two short hairpin RNA (shRNA) sequences against MYL9 were synthesized and cloned into pSUPER-retro-puro plasmids. The recombinant plasmids or negative control vector ligated by scrambled-base hairpin oligos were co-transfected with packaging plasmids pIK into 293T cells. Thereafter, the supernatant was harvested, which was utilized for infecting MGC-803 and BGC-823 cells. The above-mentioned cells were plated into 6-well plates. When the confluency reached 50%, transfections were presented with Lipofectamine 2000 (Invitrogen, United States) in accordance with the manufacturer's instructions. Following 48 h, transfection efficiencies were evaluated.

### Cell Counting Kit (CCK)-8

For the determination of viable MGC-803 and BGC-823 cells, CCK-8 (Dojindo, Japan) kits were adopted. In brief, cells were administered on a 96-well plate (3 × 10^3^ cells/well). Following cultivation lasting 24 h, 10 μl of CCK-8 reagent was added, followed by incubation at 37°C lasting 1 h. The absorbance values were tested at 450 nm with an ultraviolet spectrophotometer at diverse time points.

### Flow Cytometry

MGC-803 and BGC-823 cells were treated with propranolol lasting 24 h. Thereafter, 100 μl cell suspension was incubated with 5 μl fluorescein isothiocyanate (FITC)-Annexin V as well as 2.5 μl propidium iodide (PI) protecting from light in accordance with the manufacturer's instruction. Apoptosis was under evaluation utilizing flow cytometry on BD FACSCanto II (BD, United States). Flow cytometry was analyzed with FlowJo software.

### Statistics

All statistical analyses were conducted with R software and GraphPad Prism software. Measurement data were displayed as mean ± SD. If the variables were normally distributed, comparisons of continuous variables between two or more than two subgroups were presented through a parametric test (Student's *t*-test or ANOVA). Otherwise, a non-parametric test (Wilcoxon rank-sum test or Kruskal–Wallis test) was presented. Principal component analyses (PCAs) were used to present the dissimilarity among diverse clusters. Hazard ratio (HR) was determined with a Cox regression model utilizing a survival package. Analyses of overall survival (OS), disease-free survival (DFS), and disease-specific survival (DSS) were conducted with Kaplan–Meier method, and the log-rank test was adopted for determining the statistical difference. Pearson's or Spearman's correlation test was used for evaluating the correlation between variables. For all statistical analyses, a two-tailed *P* < 0.05 indicated significance.

## Results

### Aging-Genomic Profiles Identify Three Diverse Molecular Phenotypes of Gastric Cancer

**Figure 1:**
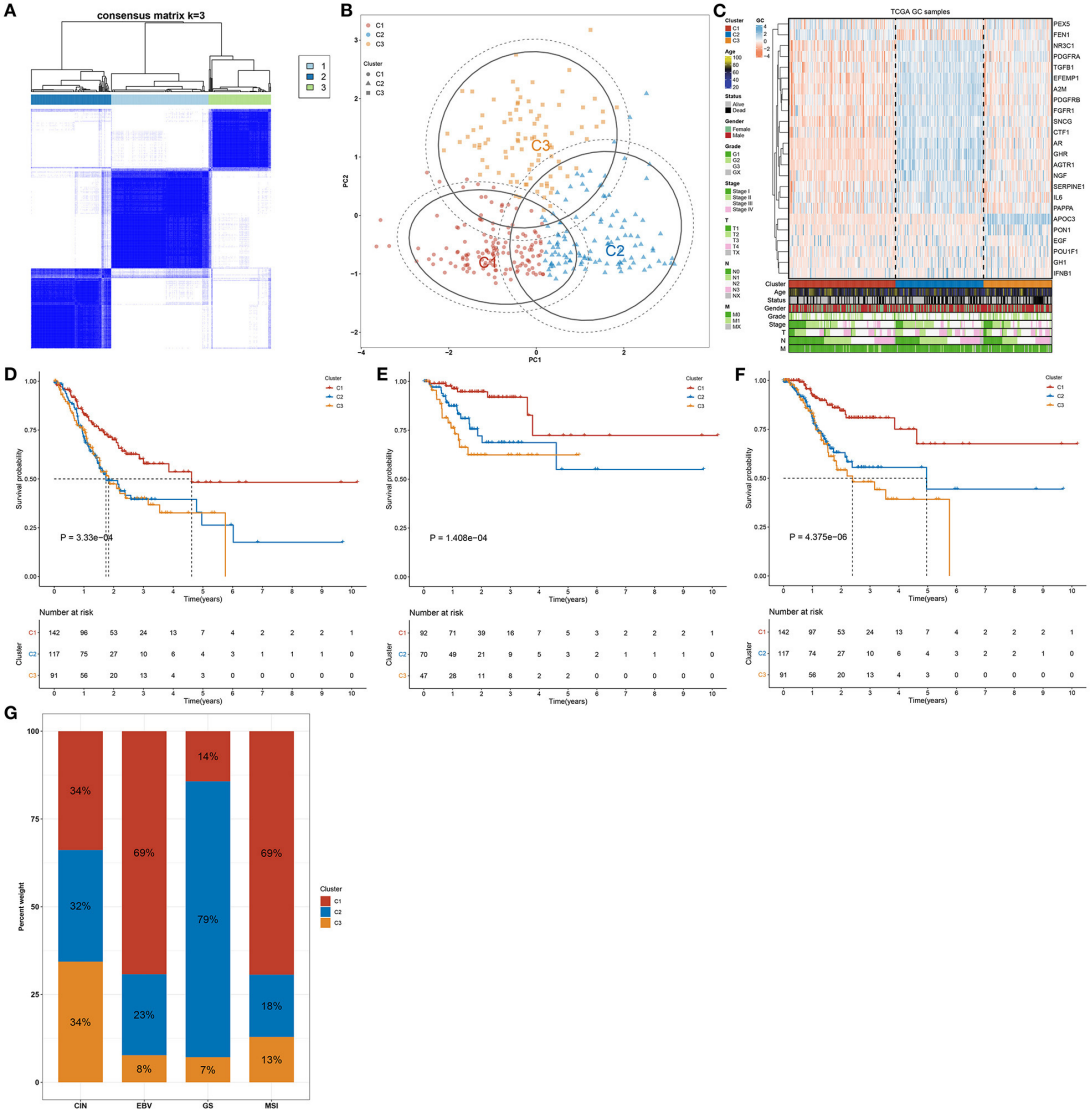
Aging-genomic profiles identify three molecular phenotypes in gastric cancer. **(A)** Heatmap depicted sample clustering at consensus *k* = 3 in accordance with the transcriptome profiling of prognostic aging-relevant genes across gastric cancer samples in the TCGA cohort. **(B)** PCA plots visualized the dissimilarity between aging-relevant molecular phenotypes. **(C)** Heatmap showed the expression patterns of prognostic aging-relevant genes in three aging-relevant molecular phenotypes. **(D–F)** Kaplan–Meier curves of **(D)** OS, **(E)** DFS, and **(F)** DSS were conducted for gastric cancer patients with diverse aging-relevant molecular phenotypes. **(G)** Distribution of aging-related molecular phenotypes C1, C2, and C3 in different molecular subtypes (CIN, EBV, GS, and MSI).

This study analyzed the expression patterns of aging-relevant genes across gastric cancer specimens in the TCGA cohort. Through univariate-cox regression analyses, abnormal expression of 24 aging-relevant genes was in relation to gastric cancer prognosis (Table 2). With the consensus clustering method, patients with gastric cancer were clustered into three aging-relevant molecular phenotypes (C1, 143 samples; C2, 117 samples; C3, 91 samples) in accordance with the transcriptome profiling of prognostic aging-relevant genes (Figure 1A). PCA uncovered the dissimilarity between aging-relevant molecular phenotypes (Figure 1B). The prominent discrepancy in expressions of prognostic aging-relevant genes was investigated among phenotypes (Figure 1C). Survival analyses demonstrated that three aging-relevant molecular phenotypes presented prominent survival outcomes. C1 phenotype possessed more favorable OS (Figure 1D), DFS (Figure 1E), and DSS (Figure 1F) outcomes than C2 and C3 phenotypes. The classification accuracy was confirmed in the GSE84437 cohort (Supplementary Figures 1A–D). Figure 1G showed the heterogeneity in the distribution of three aging-relevant molecular phenotypes (C1, C2, and C3) among the most known molecular subtypes (CIN, EBV, GS, and MSI). C1 subtype occupied the highest percentage in EBV and MSI, while C2 occupied the highest percentage in GS. Thus, the aging-relevant molecular phenotypes presented remarkable associations with the most known molecular subtypes of gastric cancer.

**Table 2:** Prognostic aging-relevant genes in gastric cancer with univariate-cox regression analyses.

**Genes**	**HR**	**HR 0.95L**	**HR 0.95H**	***P*-value**
GHR	1.5437	1.1596	2.0551	0.0029
POU1F1	3.5083	1.2136	10.142	0.0205
GH1	1.9524	1.0594	3.5981	0.0320
NGF	1.7419	1.2548	2.4180	0.0009
EGF	1.5420	1.1022	2.1572	0.0115
PDGFRB	2.2543	1.1351	4.4766	0.0202
PEX5	0.3813	0.1479	0.9831	0.0460
NR3C1	2.0259	1.1514	3.5646	0.0143
TGFB1	3.0279	1.2103	7.5751	0.0179
APOC3	1.2600	1.0596	1.4984	0.0089
AR	1.3530	1.0050	1.8215	0.0463
FEN1	0.4273	0.1849	0.9872	0.0466
A2M	2.4406	1.0382	5.7374	0.0408
SNCG	1.7807	1.2627	2.5113	0.0010
PON1	1.4702	1.0866	1.9893	0.0125
IL6	1.3283	1.0257	1.7201	0.0314
FGFR1	1.5856	1.0199	2.4652	0.0406
PAPPA	1.6353	1.1335	2.3593	0.0085
EFEMP1	1.9565	1.2875	2.9732	0.0017
AGTR1	1.2752	1.0316	1.5763	0.0246
PDGFRA	1.8412	1.1501	2.9478	0.0110

### Aging-Relevant Molecular Phenotypes With Diverse Cancer Mutational Genome

**Figure 2:**
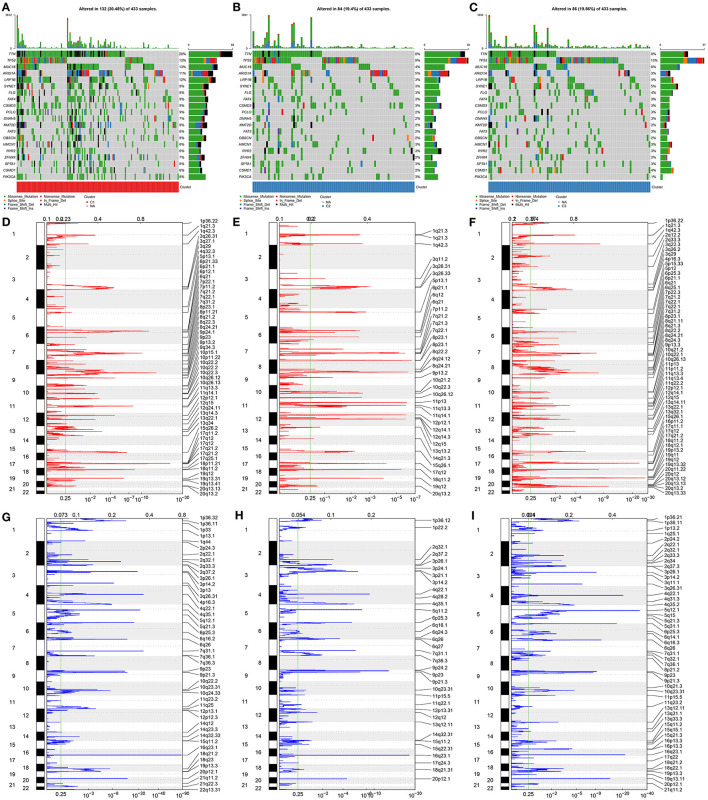
Aging-relevant molecular phenotypes with diverse cancer mutational genomes. **(A–C)** Oncoprint of somatic mutation status across patients with gastric cancer in aging-related molecular phenotypes **(A)** C1, **(B)** C2, and **(C)** C3. Individual patients were represented in each column. The right bar plots showed the mutation frequencies of the first 20 mutated genes in each molecular phenotype. **(D–F)** The amplifications of genes were separately shown in molecular phenotypes **(D)** C1, **(E)** C2, and **(F)** C3. The false-discovery rate (*q* value) and scores for amplifications were plotted against genomic locations. **(G–I)** The deletions of genes were separately shown in molecular phenotypes **(G)** C1, **(H)** C2, and **(I)** C3. The *q*-value and scores for deletions were depicted against genomic locations. Dotted lines indicated the centromeres. The green line represented 0.25 of *q*-value cutoff value that determined significance.

The preclinical studies and clinical trials have uncovered that somatic mutation is linked to therapeutic response, survival outcome, and clinical benefit of patients with gastric cancer (36). Hence, this study evaluated the distributions of somatic mutations across gastric cancer among three aging-related molecular phenotypes. We investigated that molecular phenotype C1 presented higher mutational frequency (132, 30.48%; Figure 2A) compared with C2 (84, 19.4%; Figure 2B) and C3 (86, 19.86%; Figure 2C). The first 20 genes with the highest mutational frequencies were shown in each phenotype. Gistic2.0 identified 54 amplifications in phenotype C1 (Figure 2D), 37 amplifications in phenotype C2 (Figure 2E), and 58 amplifications in phenotype C3 (Figure 2F). Meanwhile, there were 46 deletions in phenotype C1 (Figure 2G), 35 deletions in phenotype C2 (Figure 2H), and 51 deletions in phenotype C3 (Figure 2I). Collectively, aging-relevant molecular phenotypes presented diverse cancer mutational genomes.

### Aging-Associated Molecular Phenotypes With Distinct Activations of Functional Pathways and Chemotherapeutic Responses

**Figure 3:**
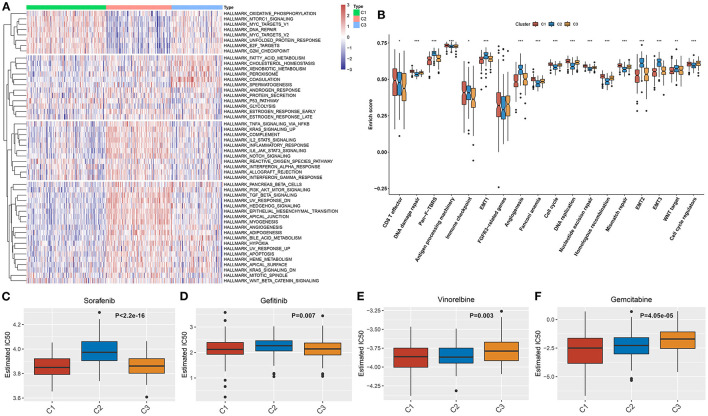
Aging-associated molecular phenotypes with distinct activations of functional pathways and chemotherapeutic responses. **(A)** Heatmap visualized the activities of the 50 hallmark gene sets in three aging-associated molecular phenotypes. **(B)** Comparisons of the activities of common biological processes among distinct molecular phenotypes. **(C–F)** Comparisons of the estimated IC50 values of chemotherapeutic agents, including **(C)** sorafenib, **(D)** gefitinib, **(E)** vinorelbine, and **(F)** gemcitabine. *p < 0.05; **p < 0.01; ***p < 0.001.

We further investigated the mechanisms underlying distinct aging-associated molecular phenotypes. In Figure 3A, we observed that immune activation pathways (complement, IL2-STAT5 signaling, inflammatory response, IL6-JAK-STAT3 signaling, allograft rejection, and interferon gamma response) and stromal activation pathways (epithelial-mesenchymal transition, angiogenesis, and WNT β-catenin signaling) were prominently upregulated in aging-associated molecular phenotype C2. Several tumorigenic pathways (mTORC1 signaling, MYC targets, DNA repair, E2F targets, and G2M checkpoint) presented a significant activation in molecular phenotypes C1 and C3. Consistently, pan-F-TBRS, immune checkpoint, EMT1-3, angiogenesis, and WNT target were prominently upregulated in molecular phenotype C2 (Figure 3B). The above data demonstrated the immune and stromal activation in molecular phenotype C2. The chemotherapeutic responses to sorafenib, gefitinib, vinorelbine, and gemcitabine were compared among three molecular phenotypes. Our results showed that molecular phenotype C2 presented the lowest therapeutic responses to sorafenib and gefitinib (Figures 3C,D) while phenotype C3 had the lowest therapeutic responses to vinorelbine and gemcitabine (Figures 3E,F).

### Aging-Associated Molecular Phenotypes Display Diverse Tumor Immune Microenvironment and Immunological Status

**Figure 4:**
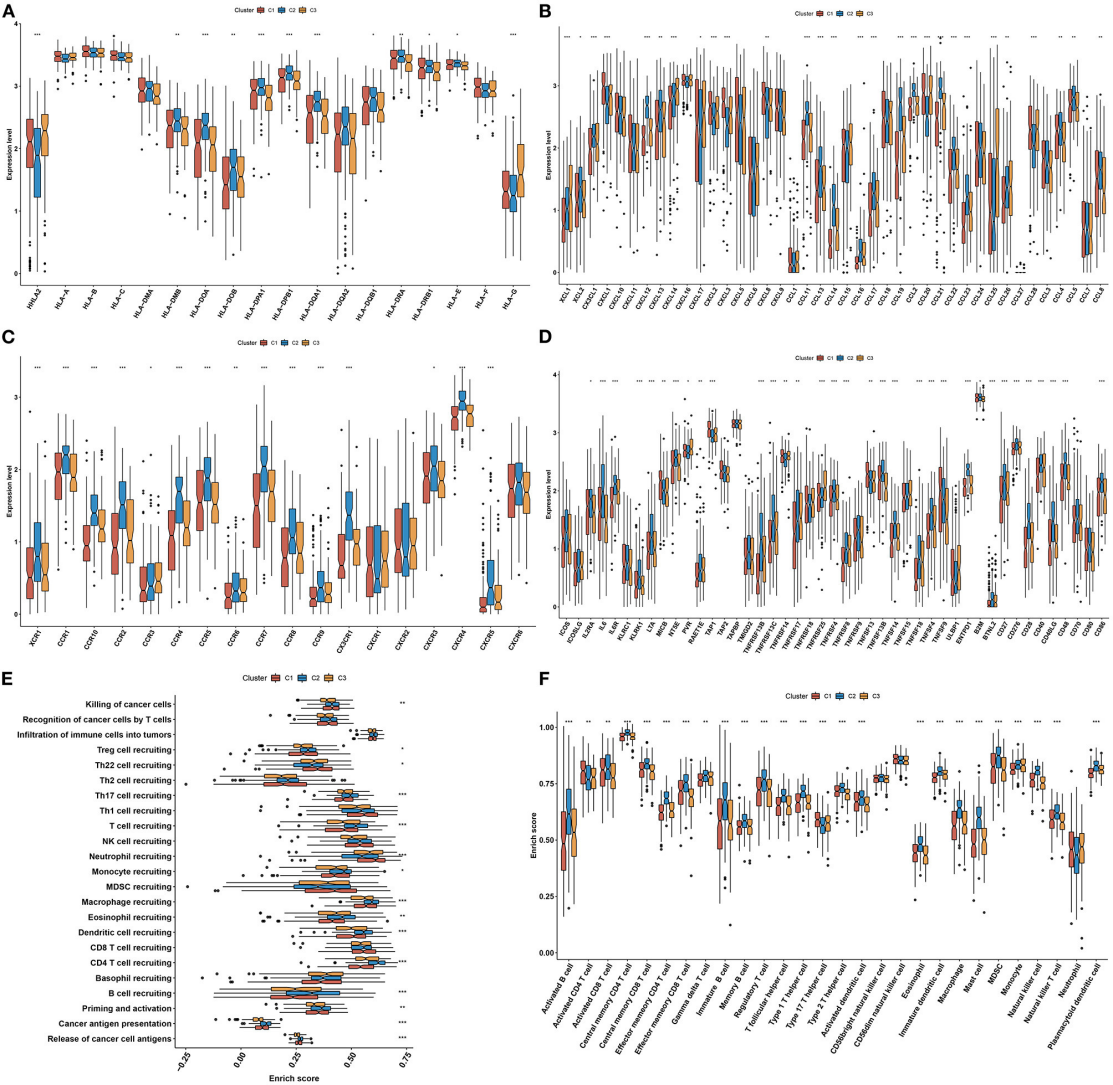
Three aging-associated molecular phenotypes display diverse tumor immune microenvironment and immunogenomic characteristics. **(A–D)** Comparisons of the mRNA expressions of immunomodulators, including **(A)** MHC, **(B)** chemokines, **(C)** receptors, and **(D)** immune checkpoint molecules among three aging-associated molecular phenotypes. **(E)** Comparisons of the activities of all steps in the cancer immunity cycle among three molecular phenotypes. **(F)** Comparisons of the infiltration levels of tumor-infiltrating lymphocytes among three molecular phenotypes. *p < 0.05; **p < 0.01; ***p < 0.001.

In Figure 4A, most MHC molecules (HLA-DMB, HLA-DOA, HLA-DOB, HLA-DPA1, HLA-DPB1, HLA-DQA1, HLA-DQB1, HLA-DRA, HLA-DRB1, and HLA-E) presented the highest expressions in aging-associated molecular phenotype C2. This was indicative that the ability of antigen presentation and processing was upregulated in phenotype C2. Additionally, molecular phenotype C2 had the highest expressions of most chemokines (XCL2, CXCL1, CXCL12, CXCL13, CCL11, CCL13, CCL14, CCL16, CCL17, CCL19, CCL2, CCL21, CCL22, CCL23, CCL4, and CCL8) and their receptors (XCR1, CCR1, CCR10, CCR2, CCR4, CCR5, CCR6, CCR7, CCR8, CCR9, CXCR1, CXCR3, CXCR4, and CXCR5), and the lowest expression of above molecules was found in molecular phenotype C1 (Figures 4B,C). Above chemokines and receptors facilitate the recruitment of effector lymphocytes like CD8+ T cell, TH17 cell, as well as antigen-presenting cells. In Figure 4D, molecular phenotype C2 was characterized by the highest expressions of most immune checkpoint molecules (IL2RA, IL6, IL6R, KLRK1, LTA, NT5E, RAET1E, TNFRSF13B, TNFRSF13C, TNFRSF17, TNFRSF4, TNFRSF8, TNFSF13B, TNFSF14, TNFSF18, TNFSF4, ENTPD1, BTNL2, CD27, CD276, CD28, CD40, CD40LG, CD48, and CD86). These data reflected the activated immunological status in molecular phenotype C2. Cancer immunity cycle activity is the overall manifestation of the chemokine system as well as immunomodulatory factors. Most steps in the cancer immunity cycle presented the highest activities in molecular phenotype C2, like cancer cell antigen release and presentation, priming and activation, recruitment of B cell, CD4 T cell, dendritic cell, eosinophil, macrophage, monocyte, T cell, Th17 cell, and Treg (Figure 4E). Thereafter, we calculated the infiltration levels of immune cells utilizing the ssGSEA algorithm. The infiltration levels of most immune cells were upregulated in molecular phenotype C2, like activated B cell, activated CD8 T cell, central memory CD4 T cell, central memory CD8 T cell, effector memory CD4 T cell, effector memory CD8 T cell, gamma delta T cell, immature B cell, memory B cell, regulatory T cell, T follicular helper cell, type 1 T helper cell, type 2 T helper cell, activated dendritic cell, eosinophil, immature dendritic cell, macrophage, mast cell, MDSC, natural killer cell, natural killer T cell, and plasmacytoid dendritic cell (Figure 4F). Collectively, molecular phenotype C2 had an inflamed tumor microenvironment.

### Aging-Associated Molecular Phenotypes Associated With Immunotherapeutic Response Predictors in Gastric Cancer

**Figure 5:**
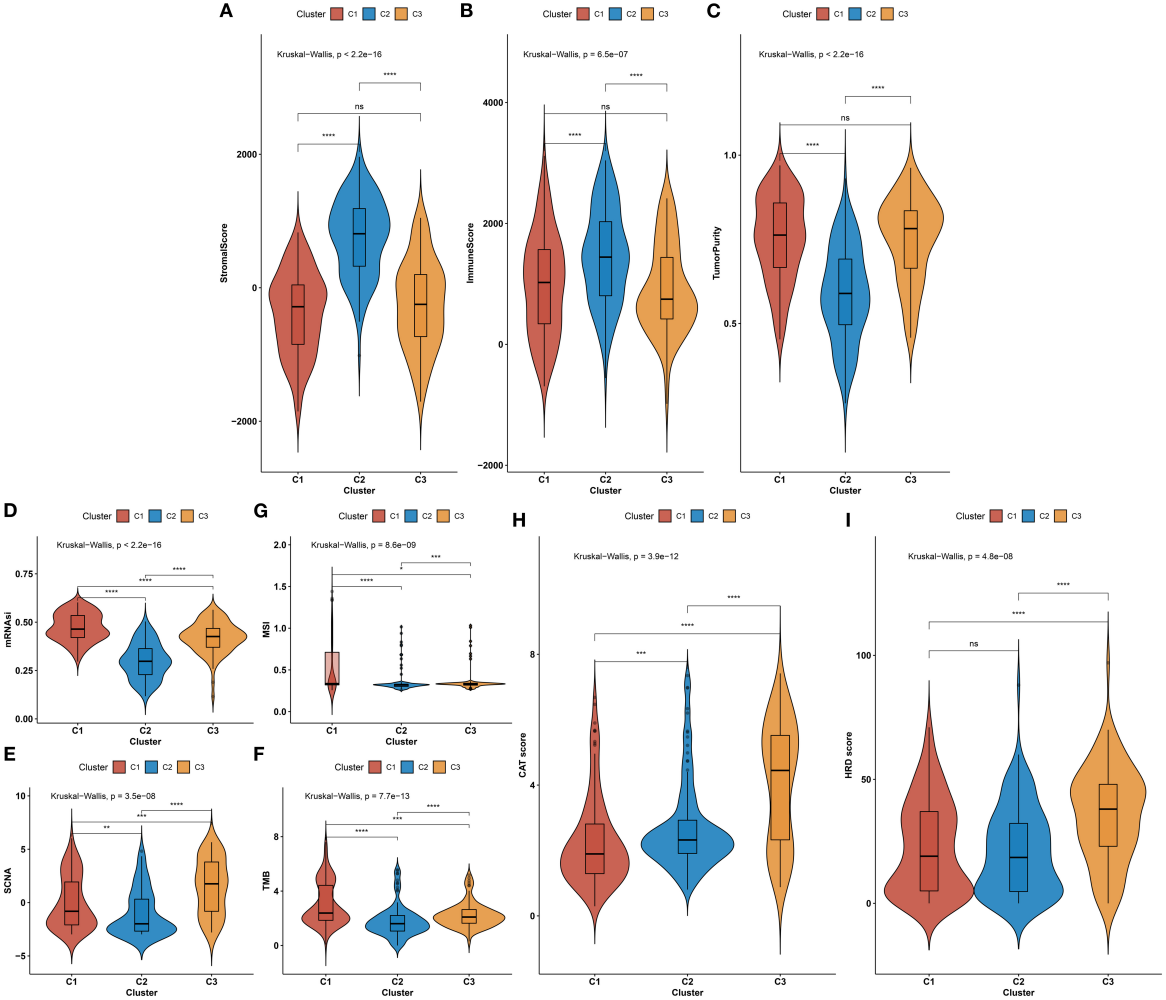
Aging-associated molecular phenotypes associated with immunotherapeutic response predictors in gastric cancer. **(A–C)** Distributions of stromal and immune scores as well as tumor purity among three aging-associated molecular phenotypes. **(D–G)** Comparisons of mRNAsi, SCNA, MSI, and TMB scores among three aging-associated molecular phenotypes. **(H,I)** Comparisons of CAT and HRD scores in diverse aging-associated molecular phenotypes. *p < 0.05; **p < 0.01; ***p < 0.001; ****p < 0.0001; ns, not significant.

We investigated the difference in immunotherapeutic responses among three aging-associated molecular phenotypes through comparisons of multiple immunotherapeutic predictors. Aging-associated molecular phenotype C2 presented higher stromal and immune scores as well as reduced tumor purity compared with C1 and C3, indicating that samples in phenotype C2 had increased infiltrations of stromal and immune cells (Figures 5A–C). The mRNAsi was quantified for reflecting the levels of cancer stem cells across gastric cancer. There was the lowest mRNAsi in phenotype C2, while the highest mRNAsi in phenotype C1 (Figure 5D). Also, we investigated the lowest SCNA in phenotype C2 but the highest SCNA in phenotype C3 (Figure 5E). In Figure 5F, phenotype C2 displayed the lowest MSI, while phenotype C1 possessed the highest MSI. Phenotype C2 presented the lowest TMB score but C1 had the highest TMB score (Figure 5G). We also evaluated the differences in cancer testis antigen (CAT) and HRD score among three aging-associated molecular phenotypes. We investigated that phenotype C3 had the highest CAT score, followed by C2 and C1 (Figure 5H). Additionally, the highest HRD score was found in phenotype C3 (Figure 5I). The above data suggested that aging-associated molecular phenotypes presented distinct immunotherapeutic responses in gastric cancer.

### Identification of Aging Molecular Phenotype-Relevant Key Genes

**Figure 6:**
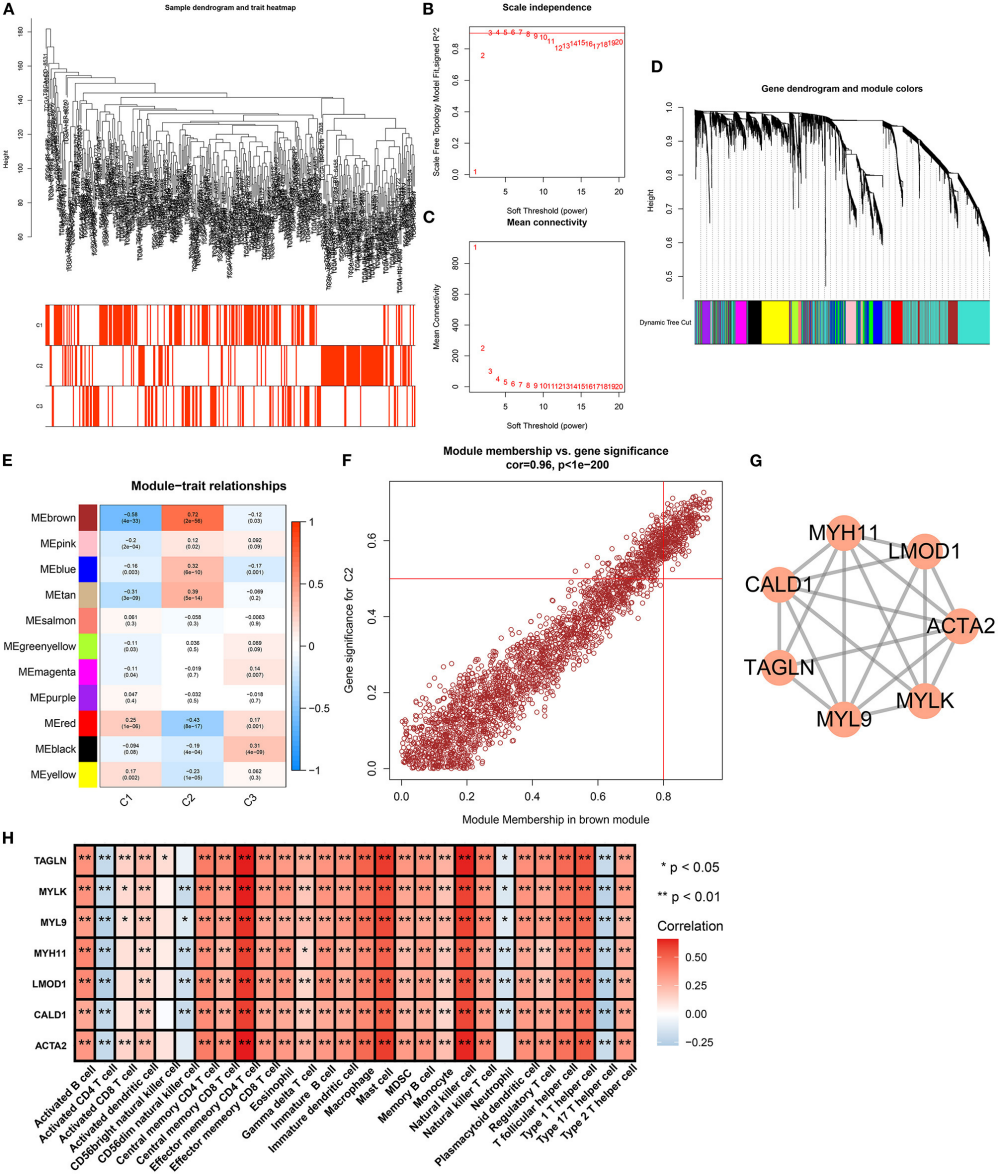
Identification of aging molecular phenotype-relevant key genes. **(A)** Sample dendrogram and heatmap were conducted based on transcriptome data of gastric cancer. The color intensity indicated aging-associated molecular phenotypes (C1, C2, and C3). **(B)** The scale-free fitting index was determined across diverse soft thresholding powers. **(C)** Mean connectivity was analyzed under different soft thresholding powers. **(D)** Clustering dendrogram was conducted on the basis of co-expression network analyses. Totally, 11 co-expression modules were merged as well as uniquely identified by diverse colors. **(E)** Heatmap showed the correlation between co-expression modules and aging-associated molecular phenotypes across gastric cancer. The brown module presented the strongest correlation module with phenotype C2. **(F)** Scatter plots depicted the interactions of module membership in the brown module with gene significance for phenotype C2. **(G)** MCODE analyses identified the most prominent module in the PPI network of genes in the brown module. **(H)** Heatmap visualized the interaction between the hub genes and the infiltration levels of immune cells. *p < 0.05; **p < 0.01.

The WGCNA method was adopted for the construction of a co-expression network as well as finding genes highly associated with aging molecular phenotypes. We first detected outliers among gastric cancer specimens on the basis of gene expression profiling. As a result, there was no outlier sample (Figure 6A). Thereafter, soft thresholding power β was calculated, and β was set at 5 for ensuring a scale-free network (Figures 6B,C). In total, 11 co-expression modules were merged, as depicted in Figure 6D. Among them, the brown module presented the strongest association with aging molecular phenotype C2 (Figure 6E). Thereafter, we evaluated intramodular analyses of genes in each module. Especially, genes in the brown module had high correlations with aging molecular phenotype C2 (Figure 6F). Eventually, 312 genes in this module were selected as aging molecular phenotype-relevant genes in accordance with the criteria of module membership >0.8 and gene significance >0.5 (Supplementary Table 3). We further observed the interactions between aging molecular phenotype-relevant genes through the STRING database. With MCODE analyses, seven aging molecular phenotype-relevant hub genes were identified, namely, ACTA2, CALD1, LMOD1, MYH11, MYL9, MYLK, and TAGLN (Figure 6G). In Figure 6H, we noted that the hub genes displayed remarkable associations with the infiltration levels of immune cells. All of them were negatively correlated to the infiltration levels of activated CD4 T cell, CD56dim natural killer cell, neutrophil, and type 17 T helper cell but were positively associated with the other immune cells.

### Verification of Prognostic Implication and Deregulated Expression of Aging Molecular Phenotype-Relevant Key Genes

**Figure 7:**
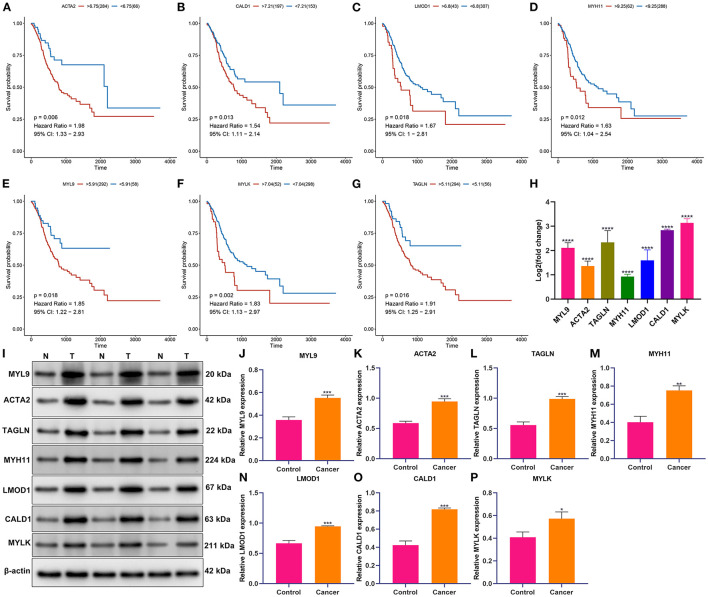
Verification of prognostic implication and deregulated expression of aging molecular phenotype-relevant key genes. **(A–G)** Kaplan–Meier curves were conducted for the investigation of survival significance of MYL9, ACTA2, TAGLN, MYH11, LMOD1, CALD1, and MYLK across patients with gastric cancer patients. **(H)** The mRNA expressions of MYL9, ACTA2, TAGLN, MYH11, LMOD1, CALD1, and MYLK were verified in 20 paired tumors and controls with RT-qPCR. The displayed graph contained normalized data to controls. **(I–P)** Western blotting was conducted for verification of the expressions of MYL9, ACTA2, TAGLN, MYH11, LMOD1, CALD1, and MYLK in tumors and controls. *p < 0.05; **p < 0.01; ***p < 0.001; ****p < 0.0001.

Survival analyses were conducted for investigations of the prognostic implications of aging molecular phenotype-relevant key genes across patients with gastric cancer. Our data demonstrated that the upregulations of ACTA2, CALD1, LMOD1, MYH11, MYL9, MYLK, and TAGLN were in relation to more dismal survival outcomes in comparisons with their downregulations (Figures 7A–G). We further verified their expressions in 20 paired tumors and controls. In Figure 7H, compared with controls, their prominent upregulations were investigated in tumors in line with RT-qPCR. Additionally, Western blotting results confirmed their abnormal expressions of these key genes in gastric cancer (Figures 7I–P).

### In Gastric Cancer Cells, MYL9 Loss Weakens Proliferation and Triggers Apoptosis

**Figure 8:**
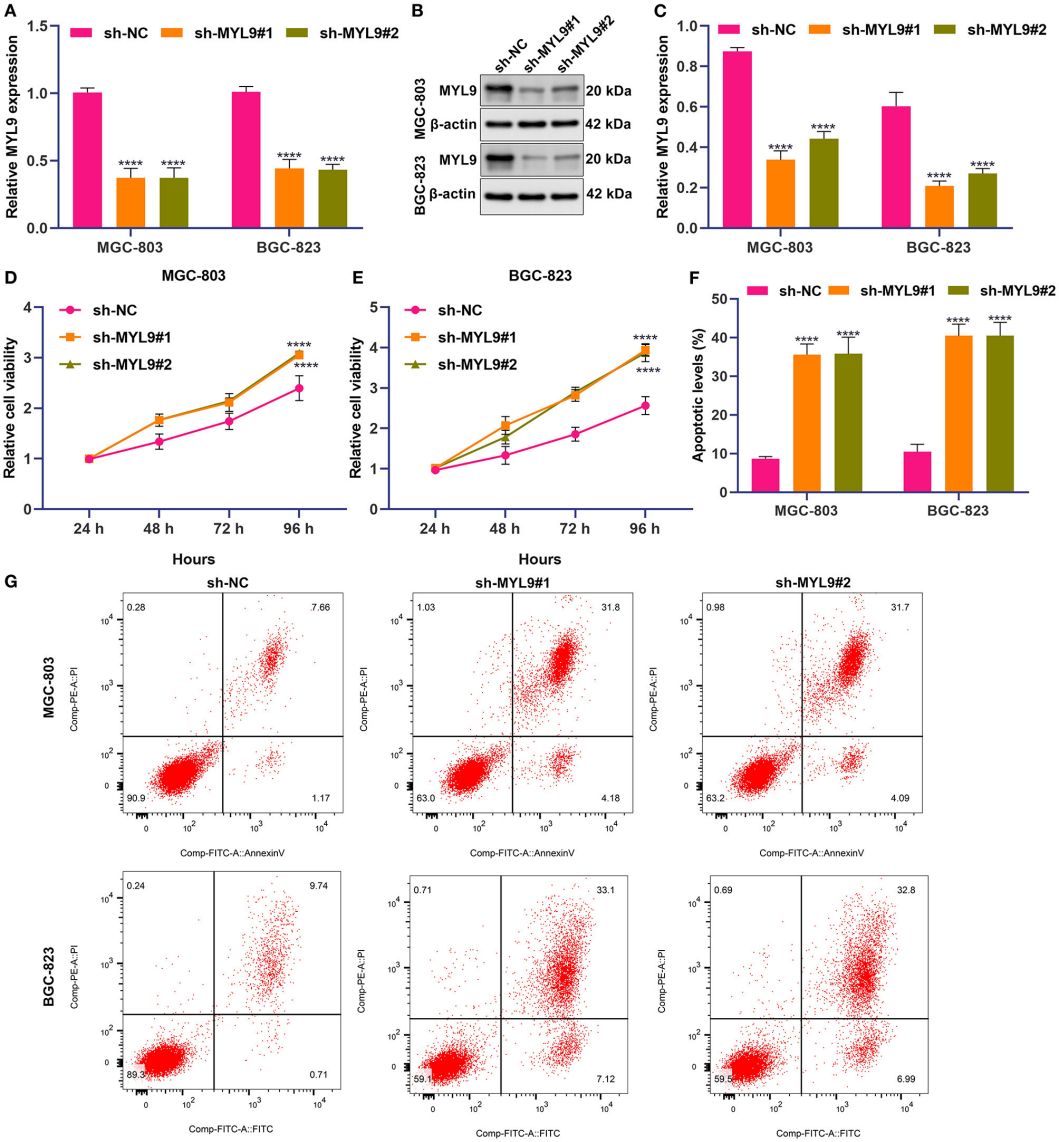
MYL9 loss weakens proliferation and triggers apoptosis in gastric cancer cells. **(A–C)** MYL9 expressions were determined in MGC-803 and BGC-823 cells under shRNAs targeting MYL9 transfections with RT-qPCR and Western blotting. **(D,E)** Viable MGC-803 and BGC-823 cells were evaluated following shRNAs against MYL9 transfections through CCK-8. **(F,G)** Apoptotic MGC-803 and BGC-823 cells were investigated after shRNAs against MYL9 transfections with flow cytometry. ****p < 0.0001.

Among aging molecular phenotype-relevant key genes, only the role of MYL9 in gastric cancer remains unknown. Thus, we investigated the function of MYL9 in gastric carcinogenesis. Herein, MYL9 expressions were reduced in MGC-803 and BGC-823 cells under two shRNAs against MYL9 transfections (Figures 8A–C). In accordance with CCK-8 results, MYL9 loss reduced the cell viability of MGC-803 and BGC-823 cells (Figures 8D,E). Additionally, apoptosis of MGC-803 and BGC-823 cells was enhanced when MYL9 expressions were defective (Figures 8F,G). The above data indicated the gastric tumorigenic roles of MYL9.

## Discussion

In our study, we conducted three aging-based molecular phenotypes with a consensus clustering algorithm. Further, aging-based molecular phenotypes were characterized by diverse clinical prognoses, mutational status as well as the immunological status of tumor microenvironment across gastric cancer. In this aspect, our findings offered individualized treatment options and prognosis evaluation for distinct subpopulations based on the aging-related molecular phenotypes.

The tumor microenvironment is comprised of a heterogeneous cellular milieu that influences cancer cell behaviors (3). The feature produces a far-reaching impact on treatment responses like immunotherapy. An inflamed tumor microenvironment combined with preexisting antitumor immunity is necessary for immunotherapy that suppresses tumor growth through tumor-cytotoxic T-cell re-invigoration. In theory, molecules and signals contribute to an inflamed tumor microenvironment that may trigger sensitivity to immunotherapy. Herein, in accordance with immunological features (immunomodulators, immune checkpoint molecules, cancer immunity cycle, and tumor-infiltrating immune cells), aging-based molecular phenotype C2 presented an inflamed tumor microenvironment. This indicated that the subpopulations in this phenotype possessed greater chances of responding to immunotherapy. Cancer stem cells contribute to chemotherapeutic resistance as well as distant metastases due to the self-renewal and tumorigenic capacities (37). Through mRNAsi, we quantified the levels of cancer stem cells across gastric cancer. There was the lowest mRNAsi in phenotype C2, while the highest mRNAsi in phenotype C1. TMB and MSI are capable of predicting the clinical responses to immunotherapy. Nevertheless, the predictors are examined utilizing complex molecular tools that are slow and expensive. Thus, it is an urgent medical requirement for developing faster and economical predictors. Our data indicated that phenotype C2 displayed the lowest MSI and TMB scores, while phenotype C1 possessed the highest MSI and TMB scores. HRD leads to impaired double-strand break repair, which is a common driving factor of carcinogenesis (38). Herein, phenotype C2 presented the features of reduced HRD score, while phenotype C3 was characterized by elevated HRD score.

Through WGCNA combined with MCODE methods, we identified seven aging molecular phenotype-relevant key genes, namely, ACTA2, CALD1, LMOD1, MYH11, MYL9, MYLK, and TAGLN. The above genes displayed the specific upregulations in gastric cancer and contributed to a dismal clinical prognosis. Previously, CALD1 acts as a prognostic indicator and also is in relation to immune infiltrates in gastric carcinoma (39). MYH11 expression is downregulated in gastric carcinoma and is indicative of a dismal clinical prognosis (40). Hypermethylation of MYLK serves as a circulating diagnostic marker of gastric carcinoma (41). Stromal fibroblasts in the microenvironment trigger gastric carcinoma metastases through the upregulation of TAGLN (42). Among them, our experimental pieces of evidence demonstrated that MYL9 deficiency reduced proliferation as well as enhanced apoptosis in gastric carcinoma cells, confirming the tumorigenic function of MYL9. Nevertheless, there were a few limitations in this study. The aging-based molecular phenotypes should be further verified in large patients from multicenter cohorts for identifying the characteristics of clinical prognosis and drug responses. Additionally, we identified aging molecular phenotype-relevant key genes, especially MYL9. Nevertheless, the specific experimental verifications should be designed for the assessment of the biological implications.

## Conclusion

Herein, our comprehensive assessment of the cellular, molecular, and genetic features correlated with aging-based molecular phenotypes generated novel insights on how gastric tumors responded to immunotherapy and guided the development of more effective combination therapeutic regimens.

## Data Availability Statement

The original contributions presented in the study are included in the article/Supplementary Material, further inquiries can be directed to the corresponding author/s.

## Ethics Statement

The study was approved by the Ethics Committee of General Hospital of Ningxia Medical University (approval no. 2020-031). The patients/participants provided their written informed consent to participate in this study.

## Author Contributions

YZhu and SY conceived and designed the study. FH, HD, and YZhou conducted most of the experiments and data analysis and wrote the manuscript. YW and JX participated in collecting data and helped to draft the manuscript. All authors reviewed and approved the manuscript.

## Funding

This work was funded by the National Key R&D Program of China (MOST-2017YFC0908300) and Ningxia Autonomous Region Key R&D Program Project (2018YBZD0418).

## Conflict of Interest

The authors declare that the research was conducted in the absence of any commercial or financial relationships that could be construed as a potential conflict of interest.

## Publisher's Note

All claims expressed in this article are solely those of the authors and do not necessarily represent those of their affiliated organizations, or those of the publisher, the editors and the reviewers. Any product that may be evaluated in this article, or claim that may be made by its manufacturer, is not guaranteed or endorsed by the publisher.

## Supplementary Material

Supplementary Figure 1 Validation of aging-genomic profiles identifies three molecular phenotypes across gastric cancer in the GSE84437 cohort. **(A)** Heatmap depicted sample clustering at consensus *k* = 3 following the transcriptome profiling of prognostic aging-relevant genes. **(B)** PCA plots confirmed the classification accuracy of aging-relevant molecular phenotypes. **(C)** Heatmap showed the expression patterns of prognostic aging-relevant genes in three aging-relevant molecular phenotypes. **(D)** Kaplan–Meier survival curves were conducted for gastric cancer patients with diverse molecular phenotypes.

Supplementary Table 1 Clinicopathological characteristics of patients with gastric cancer in TCGA and GSE84437 cohorts.

Supplementary Table 2 The list of aging-relevant genes.

Supplementary Table 3 The list of 312 genes in the brown module.

## Abbreviations

FPKM: Fragments Per Kilobase Million
TCGA: The Cancer Genome Atlas
GDC: Genomic Data Commons
GEO: Gene Expression Omnibus
TPMs: transcripts per kilobase million
TMB: Tumor mutation burden
can: copy number alteration
SCNAs: Somatic copy-number alterations
PCA: Principal component analyses
GSVA: gene set variation analysis
MSigDB: Molecular Signatures Database
Pan-F-TBRS: pan-fibroblast TGF-β response signature
EMT: epithelial-mesenchymal transition
ssGSEA: single sample gene set enrichment analysis
GDSC: Genomics of Drug Sensitivity in Cancer
IC50: half-maximal inhibitory concentration
ESTIMATE: Estimation of Stromal and Immune cells in Malignant Tumor tissues using Expression data
WGCNA: Weighted gene co-expression network analysis
STRING: Search Tool for the Retrieval of Interacting Genes/Proteins
PPI: protein-protein interaction
MCODE: Molecular Complex Detection
RT-qPCR: Real-time quantitative reverse transcription PCR.
