# Supplementary figures and images for: Depiction of Aging-Based Molecular Phenotypes With Diverse Clinical Prognosis and Immunological Features in Gastric Cancer
Source: Front Med (Lausanne). 2022 Feb 1;8:792740. doi: 10.3389/fmed.2021.792740 (PMC8843835; doi:10.3389/fmed.2021.792740)

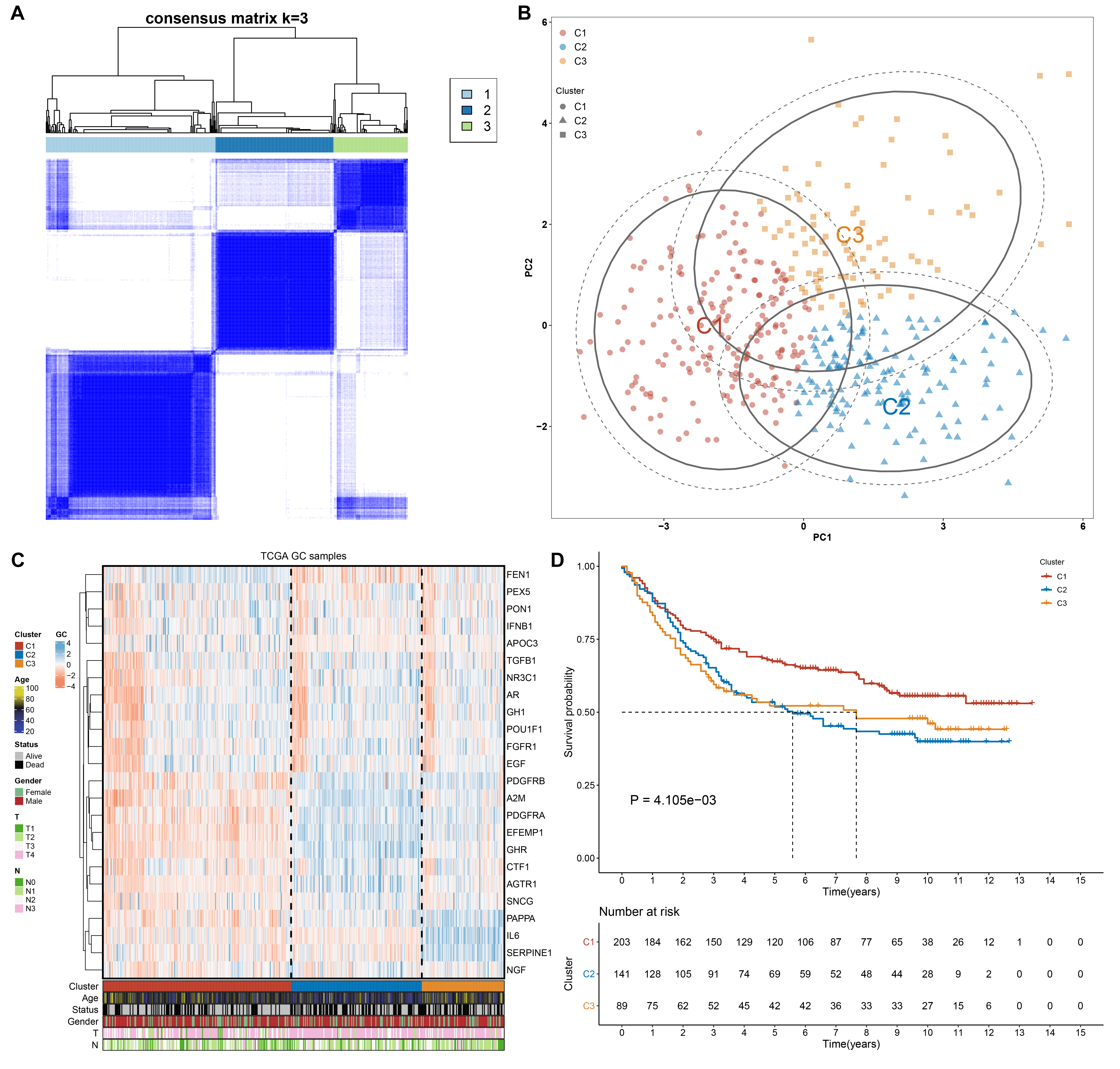

Supplement: Supplementary Figure 1 — Validation of aging-genomic profiles identifies three molecular phenotypes across gastric cancer in the GSE84437 cohort. (A) Heatmap depicted sample clustering at consensus k = 3 following the transcriptome profiling of prognostic aging-relevant genes. (B) PCA plots confirmed the classification accuracy of aging-relevant molecular phenotypes. (C) Heatmap showed the expression patterns of prognostic aging-relevant genes in three aging-relevant molecular phenotypes. (D) Kaplan–Meier survival curves were conducted for gastric cancer patients with diverse molecular phenotypes. [file Image_1.TIF]
